# *catena*-Poly[(triaqua­zinc)-μ-furan-2,5-dicarboxyl­ato-κ^3^ *O* ^2^:*O* ^2^,*O* ^2′^]

**DOI:** 10.1107/S1600536812012111

**Published:** 2012-03-31

**Authors:** Ya-Feng Li, Yue Gao, Yue Xu, Xiao-Lin Qin, Wen-Yuan Gao

**Affiliations:** a School of Chemical Engineering, Changchun University of Technology, Changchun 130012, People’s Republic of China

## Abstract

In the crystal structure of the title compound, [Zn(C_6_H_2_O_5_)(H_2_O)_3_]_*n*_, an infinite chain is formed along [001] by linking of the Zn(H_2_O)_3_ entities with one carboxyl­ate group of the furan-2,5-dicarboxyl­ate ligand. Adjacent chains are linked by O_water_—H⋯O hydrogen-bonding inter­actions. The Zn(H_2_O)_3_O_3_ polyhedron displays a distorted octa­hedral geometry with one weak Zn—O_carboxyl­ate_ coordination [2.433 (8) A°] and two water mol­ecules located in axial positions. Except for one of the axial water molecules and two adjacent H atoms, the other atoms (including H atoms) possess site symmetry *m*.

## Related literature

For background to materials with metal-organic framework structures, see: Chui *et al.* (1999 ▶); Corma *et al.* (2010 ▶); Ferey (2008 ▶); Li *et al.* (1999 ▶); Ma *et al.* (2009 ▶); Murray *et al.* (2009 ▶); Tranchemontagne *et al.* (2009 ▶). 
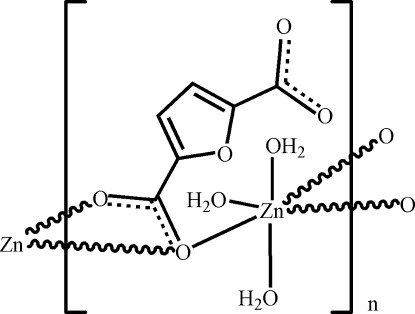


## Experimental

### 

#### Crystal data

[Zn(C_6_H_2_O_5_)(H_2_O)_3_]
*M* *_r_* = 273.51
Orthorhombic, 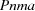

*a* = 7.3677 (15) Å
*b* = 8.1353 (16) Å
*c* = 15.107 (3) Å
*V* = 905.5 (3) Å^3^
*Z* = 4
Mo *K*α radiation
μ = 2.74 mm^−1^
*T* = 293 K
0.42 × 0.36 × 0.23 mm

#### Data collection

Rigaku R-AXIS RAPID diffractometer
Absorption correction: multi-scan (*ABSCOR*; Higashi, 1995 ▶) *T*_min_ = 0.33, *T*_max_ = 0.54
8443 measured reflections
1121 independent reflections
1029 reflections with *I* > 2σ(*I*)
*R*_int_ = 0.027

#### Refinement

*R*[*F* ^2^ > 2σ(*F* ^2^)] = 0.083
*wR*(*F* ^2^) = 0.224
*S* = 1.11
1121 reflections
98 parameters
87 restraints
H atoms treated by a mixture of independent and constrained refinement
Δρ_max_ = 2.22 e Å^−3^
Δρ_min_ = −1.90 e Å^−3^

### 

Data collection: *PROCESS-AUTO* (Rigaku, 1998 ▶); cell refinement: *PROCESS-AUTO*; data reduction: *CrystalStructure* (Rigaku/MSC, 2002) ▶; program(s) used to solve structure: *SHELXS97* (Sheldrick, 2008 ▶); program(s) used to refine structure: *SHELXL97* (Sheldrick, 2008 ▶); molecular graphics: *DIAMOND* (Brandenburg, 2000 ▶); software used to prepare material for publication: *SHELXL97*.

## Supplementary Material

Crystal structure: contains datablock(s) I, global. DOI: 10.1107/S1600536812012111/qm2058sup1.cif

Structure factors: contains datablock(s) I. DOI: 10.1107/S1600536812012111/qm2058Isup2.hkl

Additional supplementary materials:  crystallographic information; 3D view; checkCIF report

## Figures and Tables

**Table 1:** Hydrogen-bond geometry (Å, °)

*D*—H⋯*A*	*D*—H	H⋯*A*	*D*⋯*A*	*D*—H⋯*A*
O1*W*—H1*A*⋯O5^i^	0.82 (3)	2.08 (8)	2.809 (10)	147 (14)
O1*W*—H1*B*⋯O4^ii^	0.83 (3)	2.16 (5)	2.957 (11)	163 (12)
O2*W*—H2*A*⋯O4^iii^	0.82 (3)	1.83 (4)	2.648 (14)	168 (14)
O2*W*—H2*B*⋯O1^iv^	0.82 (3)	1.67 (3)	2.491 (11)	177 (15)

Symmetry codes: (i) 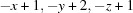; (ii) 
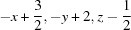
; (iii) 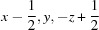; (iv) 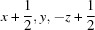.

**Fig. 1.**
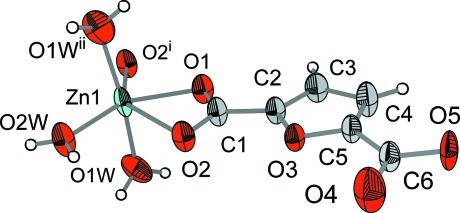
The unit cell of (I), showing the atomic labelling scheme and displacement ellipsoids at the 50% probability level. [Symmetry codes: (i) -0.5 + x, y, 0.5 - z; (ii) x, 1.5 - y, z.]

**Fig. 2.**
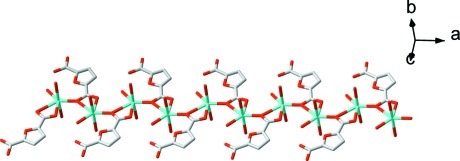
The stick plot of (I), displaying the infinite chain along [001] direction formed by linking the Zn with carboxyl of furan-2,5-dicarboxylate.

**Fig. 3.**
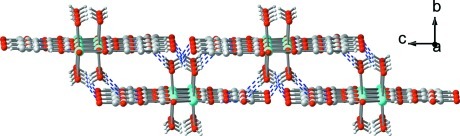
The ball-stick packing diagram of (I). The adjacent chains are holded together by the H-bonding interactions.

## supplementary crystallographic information

### Comment

During past decades, many efforts have been made to construct MOF materials due to their potential applications including gas absorption and reaction catalysis (Ma, *et al.*, 2009; Murray, *et al.*, 2009; Corma, *et al.*, 2010). Much attention has been focused on MOFs based on a phenyl ring with carboxylate groups (Chui, *et al.*, 1999; Li, *et al.*, 1999; Ferey, 2008; Tranchemontagne, *et al.*, 2009). However, 5-membered rings with carboxylate groups as described here are rarely studied. Recently, we utilized furan-2,5-dicarboxyl acid as a ligand for MOF construction. In this work, a novel chainlike compound, [Zn^.^(C_6_O_5_H_2_)^.^3H_2_O]_n_ (I), was synthesized.

The asymmetric unit of (I) is comprised of one Zn(II) cation, one furan-2,5-dicarboxylate anion and three H_2_O molecules (Fig.1). The Zn cation is coordinated by three carboxylate O atoms and three water molecules of which two are at the axial positions generating a distorted octahedron. Carboxylate oxygen O2 of (Zn—O_carboxylate_ = 2.433 (8) Å) is very weakly ligated to the Zn cation. If this interaction is excluded the Zn displays triganol bipyramid geometry but the chain property is retained. Only the O2 carboxyl of the furan-2,5-dicarboxylate is involved in the formation of the infinite chain. The carboxyl has an µ_2_:η^1^,η^2^ bonding mode.

The infinite chain of Zn cations linked by one carboxylate of furan-2,5-dicarboxylate is shown in (Fig.2). The adjacent chains are held together by H-bonding interactions of O_water_—H···O (Fig.3).

### Experimental

(I) was synthesized under solvothermal condition. In a typical preparation, furan-2,5-dicarboxyl acid (0.312 g, 2.0 mmol) and Zn(NO_3_)_2_^.^6H_2_O (0.592 g, 2.0 mmol) were dissolved in a mixture of EtOH (2.9 ml, 50 mmol) and DMF (3.9 ml, 50*m* mol) with stirring. Then, the clear solution with molar ratio of 1 (furan-2,5-dicarboxyl acid): 1 (Zn(NO_3_)_2_^.^6H_2_O): 25 (EtOH): 25 (DMF) was tranferred into a 23 ml autoclave and heated at 393 K for 24hrs. After naturally cooling to room temperature, colorless blocks were collected by filtration as a single phase.

### Refinement

Water H atoms were located in a difference Fourier map and were refined with O—H = 0.82 (2) Å, H···H = 1.37 (2) Å and *U*_iso_(H) = 1.2Ueq(O). The carbon H-atoms were placed in calculated positions (C—H = 0.93 Å) and were included in the refinement in the riding-model approximation, with *U*_iso_(H) = 1.2Ueq(C).

### Crystal data

**Table:**

[Zn(C_6_H_2_O_5_)(H_2_O)_3_]	*F*(000) = 552
*M**_r_* = 273.51	*D*_x_ = 2.006 Mg m^−^^3^
Orthorhombic, *P**n**m**a*	Mo *K*α radiation, λ = 0.71073 Å
Hall symbol: -P 2ac 2n	Cell parameters from 1000 reflections
*a* = 7.3677 (15) Å	θ = 2.8–30.2°
*b* = 8.1353 (16) Å	µ = 2.74 mm^−^^1^
*c* = 15.107 (3) Å	*T* = 293 K
*V* = 905.5 (3) Å^3^	Block, colorless
*Z* = 4	0.42 × 0.36 × 0.23 mm

### Data collection

**Table:**

Rigaku R-AXIS RAPID diffractometer	1121 independent reflections
Radiation source: fine-focus sealed tube	1029 reflections with *I* > 2σ(*I*)
Graphite monochromator	*R*_int_ = 0.027
Detector resolution: 10.00 pixels mm^-1^	θ_max_ = 30.2°, θ_min_ = 2.8°
ω scans	*h* = −10→10
Absorption correction: multi-scan (*ABSCOR*; Higashi, 1995)	*k* = −9→9
*T*_min_ = 0.33, *T*_max_ = 0.54	*l* = −19→16
8443 measured reflections	

### Refinement

**Table:**

Refinement on *F*^2^	Primary atom site location: structure-invariant direct methods
Least-squares matrix: full	Secondary atom site location: difference Fourier map
*R*[*F*^2^ > 2σ(*F*^2^)] = 0.083	Hydrogen site location: inferred from neighbouring sites
*wR*(*F*^2^) = 0.224	H atoms treated by a mixture of independent and constrained refinement
*S* = 1.11	*w* = 1/[σ^2^(*F*_o_^2^) + (0.119*P*)^2^ + 7.3441*P*] where *P* = (*F*_o_^2^ + 2*F*_c_^2^)/3
1121 reflections	(Δ/σ)_max_ < 0.001
98 parameters	Δρ_max_ = 2.22 e Å^−^^3^
87 restraints	Δρ_min_ = −1.90 e Å^−^^3^

### Special details

**Table:**

Geometry. All e.s.d.'s (except the e.s.d. in the dihedral angle between two l.s. planes) are estimated using the full covariance matrix. The cell e.s.d.'s are taken into account individually in the estimation of e.s.d.'s in distances, angles and torsion angles; correlations between e.s.d.'s in cell parameters are only used when they are defined by crystal symmetry. An approximate (isotropic) treatment of cell e.s.d.'s is used for estimating e.s.d.'s involving l.s. planes.
Refinement. Refinement of *F*^2^ against ALL reflections. The weighted *R*-factor *wR* and goodness of fit *S* are based on *F*^2^, conventional *R*-factors *R* are based on *F*, with *F* set to zero for negative *F*^2^. The threshold expression of *F*^2^ > σ(*F*^2^) is used only for calculating *R*-factors(gt) *etc*. and is not relevant to the choice of reflections for refinement. *R*-factors based on *F*^2^ are statistically about twice as large as those based on *F*, and *R*- factors based on ALL data will be even larger.

### Fractional atomic coordinates and isotropic or equivalent isotropic displacement parameters (Å^2^)

**Table:**

	*x*	*y*	*z*	*U*_iso_*/*U*_eq_	
Zn1	0.33412 (15)	0.7500	0.19775 (8)	0.0399 (6)	
O1	0.3238 (10)	0.7500	0.3352 (6)	0.042 (2)	
O2	0.5848 (11)	0.7500	0.3026 (5)	0.043 (2)	
O4	0.9702 (12)	0.7500	0.5701 (7)	0.077 (3)	
O5	0.8412 (10)	0.7500	0.6964 (5)	0.053 (3)	
C1	0.4723 (12)	0.7500	0.3584 (7)	0.038 (3)	
O3	0.6726 (9)	0.7500	0.4770 (5)	0.0339 (17)	
C2	0.5127 (13)	0.7500	0.4532 (7)	0.033 (2)	
C3	0.4138 (16)	0.7500	0.5251 (8)	0.044 (3)	
H3	0.2875	0.7500	0.5257	0.053*	
C4	0.5222 (16)	0.7500	0.6006 (8)	0.051 (3)	
H4	0.4908	0.7500	0.6603	0.062*	
C5	0.6686 (10)	0.7500	0.5654 (6)	0.035 (2)	
C6	0.8312 (12)	0.7500	0.6104 (7)	0.037 (3)	
O1W	0.3316 (10)	1.0258 (13)	0.1901 (6)	0.066 (2)	
H1A	0.313 (18)	1.075 (12)	0.237 (5)	0.079*	
H1B	0.405 (14)	1.075 (12)	0.158 (6)	0.079*	
O2W	0.5094 (11)	0.7500	0.1041 (6)	0.052 (2)	
H2A	0.513 (19)	0.7500	0.0495 (18)	0.063*	
H2B	0.612 (9)	0.7500	0.126 (8)	0.063*	

### Atomic displacement parameters (Å^2^)

**Table:**

	*U*^11^	*U*^22^	*U*^33^	*U*^12^	*U*^13^	*U*^23^
Zn1	0.0127 (6)	0.0881 (13)	0.0189 (7)	0.000	0.0014 (4)	0.000
O1	0.021 (3)	0.076 (5)	0.030 (4)	0.000	−0.004 (3)	0.000
O2	0.020 (3)	0.080 (5)	0.030 (3)	0.000	0.000 (3)	0.000
O4	0.044 (5)	0.141 (8)	0.046 (5)	0.000	0.002 (4)	0.000
O5	0.022 (4)	0.119 (10)	0.017 (4)	0.000	−0.005 (3)	0.000
C1	0.015 (4)	0.075 (7)	0.024 (4)	0.000	−0.002 (3)	0.000
O3	0.018 (3)	0.060 (4)	0.023 (3)	0.000	−0.003 (2)	0.000
C2	0.014 (3)	0.063 (7)	0.023 (4)	0.000	−0.001 (3)	0.000
C3	0.031 (4)	0.069 (6)	0.034 (4)	0.000	0.000 (4)	0.000
C4	0.027 (5)	0.101 (9)	0.027 (5)	0.000	0.000 (4)	0.000
C5	0.024 (4)	0.058 (6)	0.021 (4)	0.000	−0.001 (3)	0.000
C6	0.023 (4)	0.065 (7)	0.022 (5)	0.000	−0.001 (3)	0.000
O1W	0.042 (4)	0.083 (5)	0.072 (5)	0.001 (3)	0.032 (3)	0.009 (4)
O2W	0.020 (3)	0.109 (6)	0.028 (4)	0.000	−0.003 (3)	0.000

### Geometric parameters (Å, º)

**Table:**

Zn1—O2^i^	1.837 (8)	O3—C2	1.232 (11)
Zn1—O2W	1.916 (8)	O3—C5	1.335 (12)
Zn1—O1	2.079 (9)	C2—C3	1.308 (16)
Zn1—O1W^ii^	2.247 (10)	C3—C4	1.392 (17)
Zn1—O1W	2.247 (10)	C3—H3	0.9300
Zn1—O2	2.433 (8)	C4—C5	1.203 (15)
O1—C1	1.149 (12)	C4—H4	0.9300
O2—C1	1.182 (13)	C5—C6	1.377 (9)
O2—Zn1^iii^	1.837 (8)	O1W—H1A	0.82 (3)
O4—C6	1.191 (14)	O1W—H1B	0.83 (3)
O5—C6	1.301 (12)	O2W—H2A	0.82 (3)
C1—C2	1.464 (14)	O2W—H2B	0.82 (3)
			
O2^i^—Zn1—O2W	132.2 (4)	C2—O3—C5	105.7 (8)
O2^i^—Zn1—O1	88.1 (3)	O3—C2—C3	106.9 (10)
O2W—Zn1—O1	139.7 (3)	O3—C2—C1	118.7 (9)
O2^i^—Zn1—O1W^ii^	89.52 (19)	C3—C2—C1	134.4 (10)
O2W—Zn1—O1W^ii^	88.14 (17)	C2—C3—C4	111.1 (11)
O1—Zn1—O1W^ii^	92.9 (2)	C2—C3—H3	124.4
O2^i^—Zn1—O1W	89.52 (19)	C4—C3—H3	124.4
O2W—Zn1—O1W	88.14 (17)	C5—C4—C3	98.7 (10)
O1—Zn1—O1W	92.9 (2)	C5—C4—H4	130.6
O1W^ii^—Zn1—O1W	174.0 (5)	C3—C4—H4	130.6
O2^i^—Zn1—O2	139.54 (17)	C4—C5—O3	117.5 (9)
O2W—Zn1—O2	88.2 (3)	C4—C5—C6	124.2 (10)
O1—Zn1—O2	51.5 (3)	O3—C5—C6	118.3 (8)
O1W^ii^—Zn1—O2	92.3 (2)	O4—C6—O5	117.4 (9)
O1W—Zn1—O2	92.3 (2)	O4—C6—C5	119.7 (10)
C1—O1—Zn1	105.6 (7)	O5—C6—C5	122.8 (9)
C1—O2—Zn1^iii^	134.7 (8)	Zn1—O1W—H1A	116 (8)
C1—O2—Zn1	86.1 (6)	Zn1—O1W—H1B	120 (8)
Zn1^iii^—O2—Zn1	139.2 (4)	H1A—O1W—H1B	112 (5)
O1—C1—O2	116.8 (11)	Zn1—O2W—H2A	139 (10)
O1—C1—C2	119.4 (10)	Zn1—O2W—H2B	109 (10)
O2—C1—C2	123.8 (9)	H2A—O2W—H2B	112 (13)
			
O2^i^—Zn1—O1—C1	180.000 (1)	Zn1^iii^—O2—C1—C2	0.000 (3)
O2W—Zn1—O1—C1	0.000 (2)	Zn1—O2—C1—C2	180.000 (2)
O1W^ii^—Zn1—O1—C1	90.58 (19)	C5—O3—C2—C3	0.000 (3)
O1W—Zn1—O1—C1	−90.58 (19)	C5—O3—C2—C1	180.000 (2)
O2—Zn1—O1—C1	0.000 (1)	O1—C1—C2—O3	180.000 (2)
O2^i^—Zn1—O2—C1	0.000 (1)	O2—C1—C2—O3	0.000 (3)
O2W—Zn1—O2—C1	180.000 (1)	O1—C1—C2—C3	0.000 (4)
O1—Zn1—O2—C1	0.000 (1)	O2—C1—C2—C3	180.000 (3)
O1W^ii^—Zn1—O2—C1	−91.93 (17)	O3—C2—C3—C4	0.000 (3)
O1W—Zn1—O2—C1	91.93 (17)	C1—C2—C3—C4	180.000 (3)
O2^i^—Zn1—O2—Zn1^iii^	180.0	C2—C3—C4—C5	0.000 (3)
O2W—Zn1—O2—Zn1^iii^	0.0	C3—C4—C5—O3	0.000 (3)
O1—Zn1—O2—Zn1^iii^	180.0	C3—C4—C5—C6	180.000 (3)
O1W^ii^—Zn1—O2—Zn1^iii^	88.07 (17)	C2—O3—C5—C4	0.000 (3)
O1W—Zn1—O2—Zn1^iii^	−88.07 (17)	C2—O3—C5—C6	180.000 (2)
Zn1—O1—C1—O2	0.000 (2)	C4—C5—C6—O4	180.000 (3)
Zn1—O1—C1—C2	180.000 (2)	O3—C5—C6—O4	0.000 (3)
Zn1^iii^—O2—C1—O1	180.000 (1)	C4—C5—C6—O5	0.000 (4)
Zn1—O2—C1—O1	0.000 (1)	O3—C5—C6—O5	180.000 (2)

Symmetry codes: (i) *x*−1/2, *y*, −*z*+1/2; (ii) *x*, −*y*+3/2, *z*; (iii) *x*+1/2, *y*, −*z*+1/2.

### Hydrogen-bond geometry (Å, º)

**Table:**

*D*—H···*A*	*D*—H	H···*A*	*D*···*A*	*D*—H···*A*
O1*W*—H1*A*···O5^iv^	0.82 (3)	2.08 (8)	2.809 (10)	147 (14)
O1*W*—H1*B*···O4^v^	0.83 (3)	2.16 (5)	2.957 (11)	163 (12)
O2*W*—H2*A*···O4^i^	0.82 (3)	1.83 (4)	2.648 (14)	168 (14)
O2*W*—H2*B*···O1^iii^	0.82 (3)	1.67 (3)	2.491 (11)	177 (15)

Symmetry codes: (i) *x*−1/2, *y*, −*z*+1/2; (iii) *x*+1/2, *y*, −*z*+1/2; (iv) −*x*+1, −*y*+2, −*z*+1; (v) −*x*+3/2, −*y*+2, *z*−1/2.

## References

[bb1] Brandenburg, K. (2000). *DIAMOND* Crystal Impact GbR, Bonn, Germany.

[bb2] Chui, S. S. Y., Lo, S. M. F., Charmant, J. P. H., Orpen, A. G. & Williams, I. D. (1999). *Science*, **283**, 1148–1150. 10.1126/science.283.5405.1148 10024237

[bb3] Corma, A., Garcia, H., Xamena, F. X. L. I. (2010). *Chem. Rev.* **110**, 4606–4655. 10.1021/cr9003924 20359232

[bb4] Ferey, G. (2008). *Chem. Soc. Rev.* **37**, 191–214. 10.1039/b618320b 18197340

[bb5] Higashi, T. (1995). *ABSCOR* Rigaku Corporation, Tokyo, Japan.

[bb6] Li, H., Eddaoudi, M., O’Keeffe, M. & Yaghi, O. M. (1999). *Nature (London)*, **402**, 276–279.

[bb7] Ma, L., Abney, C. & Lin, W. (2009). *Chem. Soc. Rev.* **38**, 1248–1256. 10.1039/b807083k 19384436

[bb9] Murray, L. J., Dinca, M. & Long, J. R. (2009). *Chem. Soc. Rev.* **38**, 1294–1314. 10.1039/b802256a 19384439

[bb8] Rigaku/MSC (2002). *CrystalStructure* Rigaku/MSC, The Woodlands, Texas, USA.

[bb10] Rigaku (1998). *PROCESS-AUTO* Rigaku Corporation, Tokyo, Japan.

[bb11] Sheldrick, G. M. (2008). *Acta Cryst.* A**64**, 112–122. 10.1107/S0108767307043930 18156677

[bb12] Tranchemontagne, D. J., Mendoza-Cortes, J. L., O’Keeffe, M. & Yaghi, O. M. (2009). *Chem. Soc. Rev.* **38**, 1257–1283. 10.1039/b817735j 19384437

